# Developing an 8-Session Transdiagnostic Metacognitive Therapy Group Protocol: A Pilot Study with 6-Month Follow-Up

**DOI:** 10.1192/j.eurpsy.2026.10918

**Published:** 2026-07-31

**Authors:** K. Ozdel, M. Çelik-Korkmaz

**Affiliations:** 1 Etlik City Hospital/Psychiatry Clinic, University of Health Sciences; 2 Psychiatry Department, Etlik City Hospital, Ankara, Türkiye

## Abstract

**Introduction:**

Metacognitive Therapy (MCT) targets maladaptive metacognitions and the cognitive attentional syndrome (CAS) (Wells 2009). Transdiagnostic interventions are increasingly encouraged to address comorbidity efficiently and to improve cost-effectiveness (McEvoy et al. 2009). However, structured brief group protocols with long-term follow-up remain limited.

**Objectives:**

This pilot study developed and tested an 8-session manualized transdiagnostic group MCT protocol for adults with mixed anxiety and depressive symptoms, examining both immediate and 6-month outcomes.

**Methods:**

Twelve participants (gathered from 3 groups) completed the intervention. The protocol included detached mindfulness, attention training, and modification of maladaptive beliefs. Outcomes were assessed at baseline, post-treatment, and 6-months: Clinical Global Impressions Severity (CGI-S), Beck Depression Inventory (BDI), Beck Anxiety Inventory (BAI), CAS-1, and Outcome Rating Scale (ORS). Within-group analyses were performed with paired tests.

**Results:**

Significant pre- to post-treatment improvements were observed across all primary measures, with large to very large effect sizes (Table 1). At 6-month follow-up, reductions in depression, anxiety, and CAS, and improvements in functioning were maintained. Comparisons between post-treatment and follow-up were not significant (p = 0.191–0.949), suggesting stability of gains.

**Table 1.** Key outcomes (Mean ± SD) and effect sizes Table 1. Long description.

Measure	Pre	Post	6-mo	d (Pre–Post)
CGI-S	4.42 ± 0.51	1.58 ± 0.99	1.75 ± 1.06	2.54
BDI	22.58 ± 10.75	9.25 ± 10.22	10.08 ± 9.81	0.89
BAI	32.92 ± 11.66	15.83 ± 10.92	17.00 ± 11.21	0.86
CAS–1	934.79 ± 138.41	379.38 ± 245.25	401.67 ± 240.12	2.10
ORS	16.82 ± 6.79	33.00 ± 8.98	31.92 ± 9.45	0.86

**Table 2.** Structure of the 8-Session Transdiagnostic MCT Protocol Table 2. Long description.

Session	Key focus
Assessment	History, consent, baseline measures
1	Orientation, model introduction, detached mindfulness
2	Review, thought suppression, worry management
3	Review, attention training, danger beliefs
4	Review, consolidation of danger belief modification
5	Review, positive metacognitive beliefs, belief testing
6	Review, skill consolidation, practice
7	Review, old vs. new plan exercise
8	Review, termination, relapse plan

**Conclusions:**

An 8-session transdiagnostic MCT group protocol was feasible, well accepted, and associated with robust reductions in distress, maladaptive metacognitions, and CAS, alongside improvements in functioning. Gains were sustained at 6 months, with no significant decline or further improvement, highlighting stability of effects. Despite encouraging outcomes, the small and uncontrolled nature of this study limits generalizability. Larger randomized controlled trials are required.

**Disclosure of Interest:**

None Declared

## Long descriptions

### Table 1. Long description

The table consists of five rows of clinical measures with their corresponding Mean plus or minus S D values at Pre, Post, and 6-mo intervals, followed by the Cohen's d effect size for Pre-Post changes.

* C G I dash S: Pre 4.42 plus or minus 0.51. Post 1.58 plus or minus 0.99. 6-mo 1.75 plus or minus 1.06. d equals 2.54.

* B D I: Pre 22.58 plus or minus 10.75. Post 9.25 plus or minus 10.22. 6-mo 10.08 plus or minus 9.81. d equals 0.89.

* B A I: Pre 32.92 plus or minus 11.66. Post 15.83 plus or minus 10.92. 6-mo 17.00 plus or minus 11.21. d equals 0.86.

* C A S dash 1: Pre 934.79 plus or minus 138.41. Post 379.38 plus or minus 245.25. 6-mo 401.67 plus or minus 240.12. d equals 2.10.

* O R S: Pre 16.82 plus or minus 6.79. Post 33.00 plus or minus 8.98. 6-mo 31.92 plus or minus 9.45. d equals 0.86.

Navigate back to Table 1..

### Table 2. Long description

The table consists of two columns: Session and Key focus.

* Assessment: History, consent, baseline measures.

* Session 1: Orientation, model introduction, detached mindfulness.

* Session 2: Review, thought suppression, worry management.

* Session 3: Review, attention training, danger beliefs.

* Session 4: Review, consolidation of danger belief modification.

* Session 5: Review, positive metacognitive beliefs, belief testing.

* Session 6: Review, skill consolidation, practice.

* Session 7: Review, old vs. new plan exercise.

* Session 8: Review, termination, relapse plan.

Navigate back to Table 2..

